# Exercise efficiency impairment in metabolic myopathies

**DOI:** 10.1038/s41598-020-65770-y

**Published:** 2020-05-29

**Authors:** Jean-Baptiste Noury, Fabien Zagnoli, François Petit, Pascale Marcorelles, Fabrice Rannou

**Affiliations:** 1 Neurology Department, Neuromuscular Center, CHRU Cavale Blanche, Brest, F-29609 France; 2 Molecular Genetics Department, APHP - GH Antoine Béclère, Paris, F-92140 France; 3 Pathology Department-EA 4685 LNB, Neuromuscular Center, CHRU Morvan, Brest, F-29609 France; 4 Department of Sport Medicine and Functional Explorations-CRNH Auvergne, Clermont-Ferrand University Hospital, G. Montpied Hospital, Clermont-Ferrand, F-63000 France

**Keywords:** Energy metabolism, Neuromuscular disease

## Abstract

Metabolic myopathies are muscle disorders caused by a biochemical defect of the skeletal muscle energy system resulting in exercise intolerance. The primary aim of this research was to evaluate the oxygen cost (∆V’O_2_/∆Work-Rate) during incremental exercise in patients with metabolic myopathies as compared with patients with non-metabolic myalgia and healthy subjects. The study groups consisted of eight patients with muscle glycogenoses (one Tarui and seven McArdle diseases), seven patients with a complete and twenty-two patients with a partial myoadenylate deaminase (MAD) deficiency in muscle biopsy, five patients with a respiratory chain deficiency, seventy-three patients with exercise intolerance and normal muscle biopsy (non-metabolic myalgia), and twenty-eight healthy controls. The subjects underwent a cardiopulmonary exercise test (CPX Medgraphics) performed on a bicycle ergometer. Pulmonary V’O_2_ was measured breath-by-breath throughout the incremental test. The ∆V’O_2_/∆Work-Rate slope for exercise was determined by linear regression analysis. Lower oxygen consumption (peak percent of predicted, mean ± SD; p < 0.04, one-way ANOVA) was seen in patients with glycogenoses (62.8 ± 10.2%) and respiratory chain defects (70.8 ± 23.3%) compared to patients with non-metabolic myalgia (100.0 ± 15.9%) and control subjects (106.4 ± 23.5%). ∆V’O_2_/∆Work-Rate slope (mLO_2_.min^−1^.W^−1^) was increased in patients with MAD absent (12.6 ± 1.5), MAD decreased (11.3 ± 1.1), glycogenoses (14.0 ± 2.5), respiratory chain defects (13.1 ± 1.2), and patients with non-metabolic myalgia (11.3 ± 1.3) compared with control subjects (10.2 ± 0.7; p < 0.001, one-way ANOVA). In conclusion, patients with metabolic myopathies display an increased oxygen cost during exercise and therefore can perform less work for a given VO_2_ consumption during daily life-submaximal exercises.

## Introduction

Metabolic myopathies are muscle disorders caused by a biochemical defect of the skeletal muscle energy system, which can result in exercise intolerance. Muscle glycogenoses (glycogen storage disease, GSD) may be related to a block of glycogen breakdown (glycogenolysis), with myophosphorylase deficiency (GSD V, McArdle disease) being the most common disorder, or a block of glycolysis caused by a defect in phosphofructokinase (GSD VII, Tarui disease) or in phosphoglycerate mutase (GSD X)^1^. During exercise, production of pyruvate is therefore blunted in muscle GSDs^1,2^, which limits the mitochondrial ATP resynthesis. Mitochondrial myopathies are due to defects in respiratory chain complexes, resulting in dysfunction of phosphorylative oxidation with defective ATP production, and greater reliance on anaerobic metabolism during exercise^2–6^. Myoadenylate deaminase (MAD) deficiency is the commonest enzyme defect of skeletal muscle, caused by mutation in the *AMPD1* gene^5,7^. During intense exercise, MAD displaces the equilibrium of the myokinase reaction (2 ADP ↔ ATP + AMP) toward ATP resynthesis by converting adenosine monophosphate (AMP) into inosine monophosphate (IMP) and ammonia^5,7^.

Reduced exercise capacity in metabolic myopathies has a multifactorial origin that may involve ‘peripheral’-muscle- mechanisms, such as impairment in mitochondrial ATP regeneration via reduction in substrate delivery to the tricarboxylic acid cycle and decreased oxidative phosphorylation, but also ‘central’ mechanisms^1–12^. Muscle glycogenoses and mitochondrial myopathies are featured by an exaggerated cardiorespiratory response to exercise, including an excessive ventilation and a disproportionate increase in cardiac output relative to muscle oxygen extraction^2–5,9–12^. Furthermore, as patients with metabolic myopathies experience exertional symptoms for months or years, the relative contribution of physical deconditioning to their exercise intolerance remains unknown^4,6^. Due to these factors, reduction in peak exercise oxygen consumption (V’O_2 max_, mLO_2_.min^−1^.kg^−1^) is commonly observed in patients with metabolic myopathies^1–6,13–16^.

During submaximal activities that are typically encountered during everyday life, a loss of exercise efficiency may also account for difficulties to maintain exercise in metabolic myopathies. From a pathophysiological perspective, disproportionate increase in oxygen consumption for a given workload can contribute to exercise intolerance and premature fatigue^17^. Such increase of the oxygen cost of exercise (∆V’O_2_/∆Work-Rate) has been reported in McArdle disease^13,14^. In contrast, examination of literature in mitochondrial myopathies offers conflicting results. Some studies reported an increased^3^, and others a decreased^18–21^ ∆V’O_2_/∆Work-Rate in respiratory chain deficiencies (RCDs) as compared to matched healthy controls (Table 1). Finally, little is known about oxygen cost of exercise in myoadenylate deaminase (MAD) deficiency.

**Table 1:** Previously published ∆V’O_2_/∆Work-rate slope in metabolic myopathies.

	Control	McArdle	Respiratory Chain Deficiency
***Paridon*** ***et al***., ***1994***			
**Number**	16		5
**Age** (yrs)	14.8		12.8 ± 3.7
**Peak V’O**_**2**_ (ml.min^−1^.kg^−1^)	—		—
**∆V’O**_**2**_**/∆Work-rate slope** (mLO_2_.min^−1^.W^−1^)	9.8 ± 0.9		6.7 ± 0.8
***Taivassalo*** ***et al., 2003***			
**Number**	32		40
**Age** (yrs)	39 ± 8		37 ± 12
**Peak V’O**_**2**_ (ml.min^−1^.kg^−1^)	32 ± 7		16 ± 8
**∆V’O**_**2**_**/∆Work-rate slope** (mLO_2_.min^−1^.W^−1^)	9.6		12.9
***Jeppesen*** ***et al., 2003***			
**Number**	18		15
**Age** (yrs)	38 ± 12.7		38 ± 11.6
**Peak V’O**_**2**_ (ml.min^−1^.kg^−1^)	39.4 ± 10.5		19.7 ± 10.1
**∆V’O**_**2**_**/∆Work-rate slope** (mLO_2_.min^−1^.W^−1^)	13.9		12.5
***Heinicke*** ***et al., 2011***			
**Number**	4		5
**Age** (yrs)	34 ± 16		42 ± 17
**Peak V’O**_**2**_ (ml.min^−1^.kg^−1^)	29.0 ± 5.3		9.0 ± 2.3
**∆V’O**_**2**_**/∆Work-rate slope** (mLO_2_.min^−1^.W^−1^)	10.6		5.3
***Gimenes*** ***et al***., ***2011***			
**Number**	10		14
**Age** (yrs)	29.0 ± 7.8		35.4 ± 10.8
**Peak V’O**_**2**_ (ml.min^−1^.kg^−1^)	30.7 ± 6.0		22.3 ± 7.2
**∆V’O**_**2**_**/∆Work-rate slope** (mLO_2_.min^−1^.W^−1^)	9.9 ± 0.7		7.4 ± 1.7
***O’Dochartaigh*** ***et al***., ***2004***			
**Number**	5	5	
**Age** (yrs)	31.2 ± 5.3	32.6 ± 4.2	
**Peak V’O**_**2**_ (ml.min^−1^.kg^−1^)	28.2	16.0	
**∆V’O**_**2**_**/∆Work-rate slope** (mLO_2_.min^-1^.W^-1^)	9.7	19.8	

When ∆V’O_2_/∆Work-rate slope was unreported, oxygen cost of exercise was extrapolated by the following formula: (Peak V’O_2_ - Rest V’O_2_)/maximal Work-rate. Data are reported as mean ± SD. Peak V’O_2_, maximum oxygen consumption.

So far, case-control studies have been mostly employed, by selecting patients with a given diagnosis of metabolic myopathy, but for whom exercise intolerance may not be the chief complaint. This shortcoming is prevented by consecutive inclusion of patients consulting for exercise intolerance and exercise-induced myalgia, in an observational study design.

The primary aim of this research was therefore to evaluate the oxygen cost during incremental exercise in different metabolic myopathies compared with patients with non-metabolic myalgia and healthy subjects, using a standardized methodology in a prospective-unselected cohort.

## Results

Table 2 summarizes the baseline characteristics and the results of exercise testing for the five subgroups of patients and the Control group. Age and BMI were similar among groups (p = 0.598 and 0.265, respectively; one-way ANOVA). One-way ANOVA showed a main effect of group for exercise duration [F(5,137) = 2.94; p = 0.015], but Games-Howell post-hoc tests did not identify significant differences between groups.

**Table 2:** Anthropometric characteristics and cardiopulmonary exercise test variables in patients with metabolic myopathies and in healthy controls.

	Control	Non-metabolic myalgia	MAD Decreased	MAD Absent	Glycogenoses	Respiratory Chain Deficiency
***Anthropometric data***						
**Number (n)**	28	73	22	7	8	5
**Sex (f/m)**	13/15	20/53	14/8	4/3	5/3	2/3
**Age (years)**	40.4 ± 13.7	38.1 ± 13.1	43.0 ± 14.1	38.6 ± 18.4	39.5 ± 26.3	31.0 ± 11.2
**BMI (kg.m**^**−2**^**)**	22.7 ± 3.2	24.3 ± 4.8	23.1 ± 3.8	27.2 ± 8.7	23.4 ± 4.1	23.1 ± 8.0
***Exercise test***						
**Duration (min)**	9.9 ± 1.0	10.3 ± 1.7	9.5 ± 1.1	9.5 ± 2.4	8.7 ± 1.3	8.8 ± 1.4
**Maximal power (W)**	198.6 ± 67.3	191.3 ± 62.5	151.5 ± 65.9	133.6 ± 63.2	66.6 ± 21.1^a,b^	111.8 ± 67.6
**% Predicted maximal power**	110.6 ± 21.5	99.8 ± 18.1	95.5 ± 20.1	87.3 ± 27.3	48.4 ± 9.9^a,b,c,d^	55.8 ± 26.5^a,b,c^
**Peak V’O**_**2**_ **(ml.min**^**−1**^**.kg**^**−1**^**)**	35.1 ± 7.8	35.0 ± 10.0	29.9 ± 9.7	28.6 ± 14.7	20.1 ± 7.8^a,b^	27.8 ± 11.2
**% Predicted peak V’O**_**2**_	106.4 ± 23.5	100.0 ± 15.9	97.3 ± 15.5	91.5 ± 23.8	62.8 ± 10.2^a,b,c^	70.8 ± 23.3^a,b^
**∆V’O**_**2**_**/∆WR (mLO**_**2**_**.min**^**−1**^**.W**^**−1**^**)**	10.2 ± 0.7	11.3 ± 1.3^A^	11.3 ± 1.1^A^	12.6 ± 1.5^A^	14.0 ± 2.5^A^	13.1 ± 1.1^A^
**HRmax (beat/min)**	172.5 ± 11.5	170.6 ± 16.7	155.6 ± 19.5^A,B^	142.7 ± 23.5	164.0 ± 25.9	162.0 ± 30.1
**% Predicted HRmax**	96.3 ± 6.0	94.7 ± 11.1	88.0 ± 9.9	78.6 ± 9.8^a,b^	90.8 ± 5.1	85.7 ± 14.8
**Peak O**_**2**_ **pulse (ml/beat)**	14.4 ± 5.1	15.0 ± 4.3	12.9 ± 3.9	13.7 ± 4.6	7.1 ± 1.9^a,b^	10.6 ± 4.5
**MCR**	0.91 ± 0.21	0.92 ± 0.20	0.78 ± 0.15	0.58 ± 0.18^a,b^	1.50 ± 0.29^a,b,c,d^	1.15 ± 0.19^c,d^
**Resting RER**	0.78 ± 0.05	0.76 ± 0.05	0.75 ± 0.04	0.78 ± 0.08	0.74 ± 0.04	0.76 ± 0.03
**Peak RER**	1.27 ± 0.06 ^C,D^	1.22 ± 0.09	1.21 ± 0.07	1.11 ± 0.10	0.91 ± 0.03^A,B,C,D,E^	1.25 ± 0.12
**Peak V’**_**E**_**/V’O**_**2**_	37.6 ± 5.4	36.7 ± 5.9	37.5 ± 4.4	32.9 ± 5.6	33.1 ± 5.1	41.6 ± 13.4
**OUES**	2.4 ± 0.8	2.4 ± 0.7	1.9 ± 0.7	2.1 ± 0.7	1.4 ± 0.6	1.8 ± 0.9
**Peak V’**_**E**_**/V’CO**_**2**_	29.7 ± 4.4	30.2 ± 4.6	31.5 ± 3.4	30.1 ± 5.0	35.3 ± 6.1	35.0 ± 12.9
**V’**_**E**_**/V’CO**_**2**_ **slope**	28.3 ± 4.8	29.8 ± 6.1	30.3 ± 3.8	28.3 ± 5.5	34.6 ± 4.51	35.5 ± 18.6
**Resting lactate (mM)**	1.3 ± 0.4	1.3 ± 0.6	1.1 ± 0.5	1.3 ± 0.3	0.8 ± 0.3^A,B,D^	2.6 ± 2.4
**Peak lactate (mM)**	6.6 ± 2.1	6.0 ± 2.7 ^C,D^	4.3 ± 1.4^A,B^	3.1 ± 1.6^A,B^	0.9 ± 0.3^A,B,C,E^	5.2 ± 1.3
**Resting ammonia (µM)**	21.9 ± 11.4	25.5 ± 12.9	20.1 ± 10.5	29.0 ± 17.8	45.5 ± 25.8	21.4 ± 14.3
**Peak ammonia (µM)**	35.6 ± 18.7	43.5 ± 30.9 ^C^	28.1 ± 14.0	34.1 ± 14.8	112.5 ± 50.5^A,B,C,D,E^	31.2 ± 13.3

Data are reported as mean ± SD. BMI, Body Mass Index Peak; HRmax, maximal heart rate; Peak O_2_ pulse, oxygen pulse at peak exercise; MCR, Metabolic-Chronotropic Relationship; RER, Respiratory Exchange Ratio; OUES, Optimal Uptake Efficiency Slope; V’_E_, minute ventilation; V’O_2_, oxygen consumption; V’CO_2_, carbon dioxide production. Data were analyzed by a one-way ANOVA, followed by post-hoc Games-Howell or Scheffé’s pairwise comparison tests according to Levene’s test results for homogeneity of variance. Lower case letter superscripts (a-c) and upper case letter superscripts (A-D) represent statistical significance with Scheffé’s or Games-Howell post-hoc tests, respectively. a,A significantly different from Control (p < 0.05). b,B significantly different from Non-metabolic myalgia (p < 0.05). c,C significantly different from MAD Decreased (p < 0.04); d,D significantly different from MAD Absent (p < 0.05). E, significantly different from Respiratory Chain Deficiency (p < 0.03).

Peak power, expressed as percent predicted value, was significantly different between the six subgroups [F(5,137) = 17.61; p < 0.001, one-way ANOVA]. Post-hoc pairwise comparisons from the Scheffé test revealed that % Predicted maximal power was significantly lower in Glycogenoses compared with healthy controls (p < 0.001), Non-metabolic myalgia (p < 0.001), MAD Decreased (p < 0.001), and MAD Absent (p = 0.015) groups. % Predicted maximal power was significantly reduced in RCD group compared with Controls (p < 0.001), non-metabolic myalgia (p = 0.001), and MAD Absent (p = 0.007).

In Glycogenoses, the average peak oxygen consumption achieved was 20.1 ± 7.8 mLO_2_.min^−1^.kg^−1^, representing 62.8 ± 10.2% of the age-predicted value. The latter result was less than for Controls (p < 0.001) and Non-metabolic myalgia (p = 0.007). In mitochondrial myopathy group, Scheffé post-hoc test revealed a significant lower percentage of predicted peak V’O_2_ achieved compared to Controls (p < 0.007) and Non-metabolic myalgia (p = 0.036).

**Figure 1:**
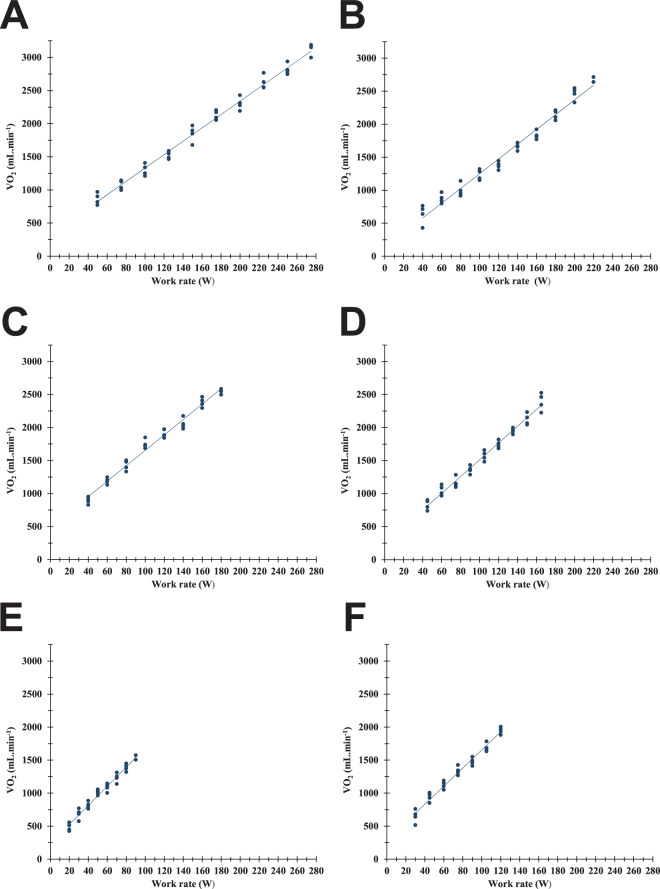
Oxygen consumption plotted against power in representative patients with metabolic myopathies. Subjects performed an incremental work test on an electronically braked bicycle until exhaustion with continuous measurement of oxygen consumption. The relationship between oxygen consumption (V’O_2_, mL.min^−1^) and work-rate (WR, watts) was determined from the 2^nd^ minute of the initial 2-min stage until the final stage that elicits peak oxygen uptake. (**A**) Control subject (male, 34 yrs; ∆V’O_2_/∆WR = 10.1 mLO_2_.min^−1^.W^−1^, R^2^ = 0.99). (**B**) Non-metabolic myalgia patient (male, 24 yrs; ∆V’O_2_/∆WR = 11.2 mLO_2_.min^−1^.W^−1^, R^2^ = 0.98). (**C**) MAD Decreased patient (male, 17 yrs; ∆V’O_2_/∆WR = 11.7 mLO_2_.min^−1^.W^−1^, R^2^ = 0.98). (**D**) MAD Absent patient (male, 16 yrs; ∆V’O_2_/∆WR = 12.7 mLO_2_.min^−1^.W^−1^, R^2^ = 0.98). (**E**) McArdle patient (male, 21 yrs; ∆V’O_2_/∆WR = 14.5 mLO_2_.min^−1^.W^−1^, R^2^ = 0.97). (**F**) Respiratory chain deficiency patient (MELAS; male, 41 yrs; ∆V’O_2_/∆WR = 13.7 mLO_2_.min^−1^.W^−1^, R^2^ = 0.97).

**Figure 2:**
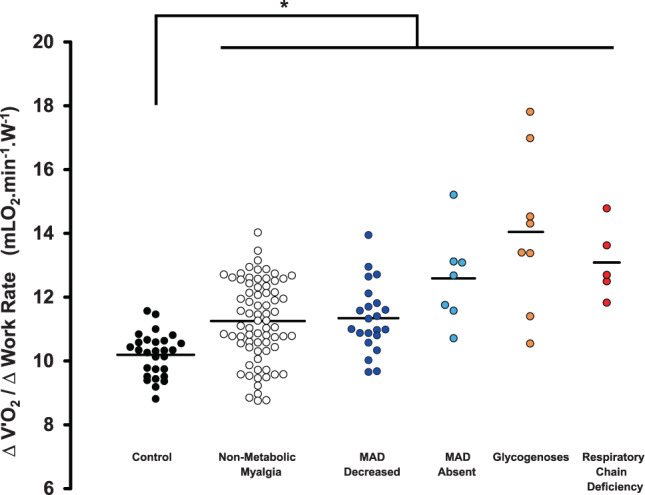
Oxygen cost of exercise in metabolic myopathies. Lines indicate the mean values. Data were analyzed using a one-way ANOVA with post hoc Games-Howell test for intergroup analysis. *Significantly different from Control (p < 0.04).

Figure 1 displays representative oxygen uptake vs. work-rate plots for each subgroup. The individual ∆V’O_2_/∆WR values in the different groups are shown in Fig. 2. The V’O_2_/WR slope was greater in all patients than in controls (p < 0.001, one-way ANOVA followed by Games-Howell post-hoc tests). V’O_2_/WR slope was similarly increased in both MAD Decreased and Non-metabolic myalgia groups compared with normal healthy subjects (11.3 ± 1.1 and 11.3 ± 1.3 vs. 10.2 ± 0.7 mLO_2_.min^−1^.W^−1^, respectively; p < 0.006).

% Predicted maximal heart rate (HRmax) was significantly lower in MAD Absent group compared with Control (p = 0.005) and Non-metabolic myalgia (p = 0.007) groups. In Glycogenoses, peak O_2_ pulse was significantly decreased compared with Control (p = 0.005) and Non-metabolic myalgia (p < 0.001) groups. The metabolic-chronotropic relationship (MCR) in the MAD Absent group (0.58 ± 0.18) was significantly lower than in the Control (p = 0.01), Non-metabolic myalgia (p = 0.003), Glycogenose (p < 0.001), and RCD (p < 0.001) groups. Conversely, the MCR of Glycogenoses (1.50 ± 0.29) was significantly higher than the MCRs of all other groups, except the RCD group (1.15 ± 0.19, p = 0.091).

The respiratory exchange ratio (RER) was similar between groups at rest, and was found to be significantly less in Glycogenoses at peak exercise (0.91 ± 0.03) compared with all other groups (p < 0.02). Control group demonstrated a significantly higher RER at peak exercise (1.27 ± 0.06) than other groups (p < 0.04) with the exception of RCD group (1.25 ± 0.12, p = 0.997).

Peak V’_E_/V’O_2_ and V’_E_/V’CO_2_ slope were not significantly different between groups (p = 0.075 and 0.059, respectively; one-way ANOVA). Post-hoc tests for oxygen uptake efficiency slope (OUES) and peak V’_E_/V’CO_2_ revealed no significant difference between groups.

Lactate concentration at rest was significantly lower in Glycogenoses (0.8 ± 0.3 mM) compared with Control (p = 0.01), Non-metabolic myalgia (p = 0.002), and MAD Absent (p = 0.042) groups. At peak exercise, lactate was significantly lower in Glycogenoses (0.9 ± 0.3 mM) compared with all other groups with the exception of MAD Absent (3.1 ± 1.6 mM, p = 0.72). Patients with absence of MAD activity showed lower lactate concentration at peak exercise compared with Control (p = 0.011) and Non-metabolic myalgia (p = 0.016) groups. Ammonia concentration at peak exercise, was significantly higher in Glycogenoses (112.5 ± 50.5 µM) compared with all other groups (p < 0.05).

## Discussion

This study provides evidence that patients with metabolic myopathies have an increased oxygen cost of exercise during an incremental test. Oxygen cost of exercise has been little studied in different metabolic myopathies in the same study^12,22^, with exercise testing performed and reported in a standardized fashion. The present prospective methodology permits comparison both across different metabolic myopathies and with non-metabolic myalgia during incremental exercise.

Visual inspection of the plots in Fig. 2 reveals two distinct profiles in patients. A first group of metabolic myopathies that includes MAD absent, glycogenoses, and mitochondrial myopathy exhibits a large increase in ∆V’O_2_/∆WR slope (12.4–14.2 mLO_2_.min^−1^.W^−1^, 95%CI). A second profile includes MAD decreased and non-metabolic myalgia, with moderate increase in mean ∆V’O_2_/∆WR slope. Nevertheless, due to substantial overlap in ∆V’O_2_/∆WR slope values between groups, post-hoc tests failed to detect statistical differences between subgroups of patients.

We observed a higher ∆V’O_2_/∆Work-Rate slope in mitochondrial myopathy in agreement with the work by Taivassalo *et al*^2^. involving forty RCD patients. In contrast, other studies reported lower ∆V’O_2_/∆Work-Rate slope in mitochondrial myopathy as compared to healthy controls^18–21^. Discrepancy among studies may originate from differences in patient respiratory-chain enzyme defects and degree of mitochondrial dysfunction in skeletal muscle (‘heteroplasmy level‘)^3,4^, as well as in the protocol of exercise testing (e.g., size of work-rate increments^23,24^). The prospective methodology we used may also account for this inconsistency, since our population of RCD patients was unselected in contrast with previous reports. In the present study, impairment of exercise capacity in RCD patients was moderate (average peak oxygen consumption = 27.8 ± 11.2 mLO_2_.min^−1^.kg^−1^; 70% predicted value) compared with previous reports^3,19–21^. Reasons for the disparity in ∆V’O_2_/∆Work-Rate slope in RCDs among studies deserve further investigation.

We observed non-significant increases in ventilation relative to oxygen consumption (peak V’_E_/V’O_2_) in RCDs and relative to carbon dioxide production (V’_E_/V’CO_2_; peak and slope values) in both RCD and McArdle patients, in keeping with previous reports^3,11,13,20,21^. The increased work by respiratory muscles in patients with glycogenoses and RCD requires higher oxygen consumption to maintain this activity and may contribute to increase the ∆V’O_2_/∆Work-Rate slope.

In McArdle patients, exercise-induced hyperammonemia has been proposed as a mechanism for the exaggerated ventilatory response to exercise^13,25^. We observed similar lactate levels at peak exercise in RCD and Control subjects, in spite of the two-fold difference in the maximal achieved power. Increased dependence on anaerobic glycolysis, with excessive lactic acidosis for a given workload, may explain in part hyperpnoea during exercise in RCDs^3,21^.

In McArdle disease, the greater reliance on lipid utilization has also been proposed as an explanatory mechanism for increased ∆V’O_2_/∆Work-Rate slope^13^, due to the lower ATP produced/oxygen consumed ratio when lipid is the substrate.

The heart rate increase for a given increase in oxygen consumption (MCR) was higher in glycogenoses, while RCDs tended to exhibit a similar pattern. McArdle disease and mitochondrial myopathy are characterized by a hyperkinetic circulatory response to exercise^2,3,5,8^, i.e. a mismatch between oxygen utilization and delivery. Despite a lower muscle oxygen extraction during exercise, resulting in a reduced oxygen arteriovenous difference, glycogenose and RCD patients demonstrate an exacerbated increase in cardiac output and tachycardia^2,3,5,8^. This excessive increase in heart rate may contribute to elevate myocardial oxygen consumption and overall oxygen uptake during exercise in RCDs and McArdle patients^13,26^. A common potential mechanism for hypercirculatory responses to exercise in RCD and McArdle patients is an increased sympathetic activition, as higher epinephrine and norepinephrine levels have been reported in these myopathies^13,27^.

In McArdle patients, it has been shown that this exaggerated heart rate increase is reduced when subjects exercise after a prolonged warm-up^2^, or after a short recovery period (‘second-wind’ phenomenon)^10^. Due to the prospective design of our study, a single –standardized- incremental exercise protocol involving a 2-min warm-up was used in all subjects. Thus, the extent to which the ‘second-wind’ mechanism contribute to the increased MCR and the greater ∆V’O_2_/∆Work-Rate slope in glycogenoses remains unknown.

In contrast to glycogenoses, the MCR was decreased in MAD Absent compared with non-metabolic myalgia patients and controls, probably due to an increased adenosine formation in this enzyme defect^7^. In MAD deficiency, AMP accumulation activates an alternate catabolic pathway that leads to excess production of adenosine^7^, which in turn produces negative chronotropic effect^28,29^. Tachycardia, with excessive myocardial oxygen consumption, and ammonia-induced hyperventilation, can be ruled out as explanatory factors for the higher ∆V’O_2_/∆Work-Rate slope observed in MAD defect. ‘Peripheral’ (muscle) mechanisms account for a greater extent to the loss of exercise efficiency in subjects with MAD defect, in contrast to RCD and glycogenose patients for whom ‘central’ factors (i.e., hyperventilation and hypercirculatory responses) are conspicuous. In MAD deficient subject, an elongation of muscle half-relaxation time has been reported following repetitive voluntary isometric contractions of the quadriceps^30^. During dynamic task, slowing of muscle relaxation promotes simultaneous contractions of antagonistic pairs and raises internal mechanical work, leading to increase the energy cost of exercise^17^. Although the reduction of peak oxygen consumption is moderate in MAD Absent patients, their greater ∆V’O_2_/∆Work-Rate slope may reduce exercise tolerance by increasing perceived effort.

Interestingly, patients with non-metabolic myalgia also exhibit a higher ∆V’O_2_/∆Work-Rate slope compared with healthy controls. A metabolic impairment has been excluded in this subgroup by an exhaustive and standardized diagnostic work-up, including a muscle biopsy^15,16,31^. It has been shown that some dystrophinopathies can present with a pseudometabolic pattern^32,33^, i.e. exercise intolerance and exercise-induced myalgia. This group displays a broad range of ∆V’O_2_/∆Work-Rate slopes, and encompasses a large population of patients with undefined genetic diagnosis. Future utilization of whole exome sequencing in this subgroup of patients with unknown myopathy will be helpful for identifying genetic mutations that underlie exercise-induced myalgia and rhabdomyolysis^33^.

From a mechanistic perspective, we acknowledge the limitation that O_2_ cost of exercise was not measured during constant exercise^22,34^. Measurement of oxygen consumption during prolonged, unloaded and loaded, steady-state exercises may be useful to determine the relative contribution of the different components of O_2_ cost of exercise through calculation of various efficiency indices (gross, net, and work efficiency as defined by Gaesser & Brooks^34^). For example, unloaded exercise is useful to determine the oxygen cost of moving the legs against zero resistance. Moreover, epinephrine and norepinephrine levels were not measured in the present study. Additional measurement of plasma catecholamine levels in all subgroups of subjects could have provided valuable insights into the underlying mechanisms of increased oxygen cost of exercise^13^.

## Conclusion

Patients with metabolic myopathies exhibit a greater oxygen cost of exercise. The clinical implication of this exacerbated oxygen cost of power production is that a patient with metabolic myopathy can perform less work for a given VO_2_ consumption during daily life-submaximal exercises. This loss of efficiency could partially explain difficulties to maintain exercise in metabolic myopathies owing to increased perceived effort.

## Materials and Methods

Written informed consent was obtained from each participant. All procedures conformed to the standards set by the Declaration of Helsinki and the protocol was approved by the institutional ethics committee of Brest Medical University Hospital (Clinical Trial NCT02362685).

### Subjects

From December 1, 2008 to November 30, 2018, all patients undergoing exercise testing for exercise intolerance and myalgia at an academic tertiary referral center were prospectively included. Glycogenoses subgroup was composed of 8 patients. One patient had Tarui disease (GSD VII), showed severe reduction of phosphofructokinase activity in muscle biopsy (0.6 U/g, normal range 19–41) and harbored a compound heterozygous mutation, IVS15 + 1 G > T and c.76 G > C, in the *PFKM* gene (see Drouet *et al*.,^35^ for details). Seven patients had McArdle disease (GSD V) with the absence of myophosphorylase activity in muscle biopsy (n = 6 patients) and confirmed in all patients by identification of pathogenic mutations in *PYGM* gene (c.148 C > T/c.148 C > T, n = 5; c.148 C > T/c.1466 C > G, n = 1; c.148 C > T/c.2262delA, n = 1). In this subgroup, the non-ischemic forearm exercise test showed a characteristic flat venous lactate curve with exaggerated increase in blood ammonia^1,5,11^.

MAD defects patients were identified by semi-quantitative histochemical analysis of fresh-frozen muscle biopsies using Fishbein’s medium^36^, as previously described^15,16,37^. Myoadenylate deaminase staining was absent in seven patients and decreased in twenty-two subjects.

Respiratory chain deficiency (RCD) group (n = 5) consisted of two patients with MELAS syndrome (m.3243 A > G *MTTL1* gene mutations), and three patients with characteristic abnormalities of mitochondrial cytopathy (ragged-red and COX-negative fibers) and decreased complex IV activity in muscle biopsy specimen.

Seventy-three patients consecutively referred for exercise-testing to investigate exercise-induced myalgia, and with no diagnosis despite extensive diagnostic work-up (including muscle biopsy)^15,16,31,37,38^, were recruited as a disease-control group (non-metabolic myalgia).

A group of 28 healthy subjects (nurses, n = 16; scuba divers, n = 12), referred for fitness for work evaluation in our department during the study period, was used as healthy control population.

### Incremental exercise test

The subjects were instructed to report to the laboratory in a fasted state, having completed no strenuous exercise within the previous 24 h. Participants attended the laboratory to complete an incremental step test on an electromagnetically braked cycle ergometer (Ergoline GmbH, Bitz, Germany) at 60 rev.min^−1^, according to an individualized protocol designed to allow patients to reach maximum exercise within the desirable range of 8 to 12 minutes. The predicted peak power output for each subject (predicted PPO) was calculated using published formulae^39^. This theoretical maximal power was adjusted according to exercise intolerance^40^, in order to obtain an estimated peak power output (estimated PPO). The progressive test consisted of cycling at 20% estimated PPO during an initial 2-min stage, followed by a stepwise increase in power (10% estimated PPO) every minute to the limit of tolerance. Expired air was analyzed breath-by-breath using an online system (Ultima Series, MedGraphics, USA), and averaged over 15 s intervals^13,18,20^. The air flow was calibrated before each test using 3-liter syringes (Hans Rudolph Inc., Kansas City, MO). Oxygen (O_2_) and carbon dioxide (CO_2_) concentrations were analyzed via galvanic fuel cell and nondispersive infrared cell, respectively. Before each test, the analyzers were calibrated using ambient air, and a gas of known O_2_ (16.00 ± 0.04%) and CO_2_ (5.00 ± 0.10%) concentrations (Air Liquide Healthcare, Plumsteadville, PA). The cycloergometer was calibrated mechanically twice a year according to the manufacturer instructions. Physiological calibration of the cycle ergometer and the gas exchange analysis system was assessed by testing, at least once a year, laboratory staff members (n = 4 to 5) using incremental and submaximal-constant work rate protocols. Calibration was further checked indirectly by annual visit of the healthy subjects for their fitness-for-work evaluation. Maximal oxygen consumption was expressed as a ratio standard with body mass (mL/min/kg) and as a percentage of theoretical value expected for sex, age, and anthropometry^24^. The slope of the relationship between the change in V’O_2_ (∆V’O_2_) for a given change in work rate (∆WR) was determined using linear regression from the 2^nd^ minute of the initial stage to peak exercise (Fig. 1). Data from the 1^st^ minute of loaded exercise were discarded from analysis, owing to the initial –exponential- delay of VO_2_ increase at the onset of work.

The following cardio-pulmonary exercise testing variables were examined: resting and peak RER, peak V’_E_/V’O_2_, peak V’_E_/V’CO_2_. The oxygen uptake efficiency slope (OUES) was derived from the linear relation of oxygen uptake (V’O_2_) vs. the logarithm of ventilation (V’_E_) throughout exercise, i.e. V’O_2_ = a log_10_ V’_E_ + b, where a is the OUES^41^. The ventilatory efficiency slope (V’_E_/V’CO_2_ slope) was calculated via least-squares linear regression (V’CO_2_ = a V’_E_ + b, where a is the V’_E_/V’CO_2_ slope)^42^. Maximal heart rate (HRmax) was measured from continuous 12-lead ECG and expressed as a percentage of the subject theoretical value (i.e., 220-age). Peak oxygen pulse was calculated by dividing peak V’O_2_ by HRmax and expressed in mL/beat. To assess the relation between heart rate and oxygen consumption during exercise, the metabolic-chronotropic relationship (MCR) was calculated as the regression line slope between percent heart rate reserve [100 * (HR_stage_ – resting heart rate)/(220 – age – resting heart rate)] and percent metabolic reserve [100 * (V’O_2 stage_ – V’O_2 rest_)/(V’O_2 peak_ – V’O_2 rest_)]^43^.

Blood sample for analysis of lactate and ammonia was drawn at rest and peak exercise from an indwelling catheter in an antecubital vein. Lactate and ammonia concentration were measured spectrophotometrically immediately following sampling^16^.

### Statistics

All statistical analyses were conducted two-tailed with α set at 0.05 and were computed using SPSS (V.25; IBM SPSS Statistics, Chicago, IL, USA). Assessment of data normality was determined by Shapiro–Wilk test. Between-group differences were assessed using One-way ANOVA followed by Scheffe’ post hoc test if group variances were homogeneous (Levene test statistic). If the result of the Levene’ test of homogeneity of variances was significant, the Games-Howell post-hoc analysis was performed to locate pairwise differences. Data are reported as mean ± SD.
